# Supplementary material for: Heparin-based hydrogel scaffolding alters the transcriptomic profile and increases the chemoresistance of MDA-MB-231 triple-negative breast cancer cells
Source: Biomater Sci. 2020 Feb 13;8(10):2786–96. doi: 10.1039/c9bm01481k (PMC7497406; doi:10.1039/c9bm01481k)
Supplement: Supplementary file 2 [file BM-008-C9BM01481K-s002.zip › Supplementary File 4/HP-B hydrogel + EGF (s)vControl/Pathways/my_analysis.Gsea.1545201170987/HALLMARK_IL6_JAK_STAT3_SIGNALING.html]

Details for gene set HALLMARK\_IL6\_JAK\_STAT3\_SIGNALING[GSEA]

|  || Dataset | expr.class.cls#HYDEGF\_versus\_CONTROL.class.cls#HYDEGF\_versus\_CONTROL\_repos |
| Phenotype | class.cls#HYDEGF\_versus\_CONTROL\_repos |
| Upregulated in class | CONTROL |
| GeneSet | HALLMARK\_IL6\_JAK\_STAT3\_SIGNALING |
| Enrichment Score (ES) | -0.5358095 |
| Normalized Enrichment Score (NES) | -2.1464524 |
| Nominal p-value | 0.0 |
| FDR q-value | 0.0 |
| FWER p-Value | 0.0 |
Table: GSEA Results Summary

  

Fig 1: Enrichment plot: HALLMARK\_IL6\_JAK\_STAT3\_SIGNALING      
 Profile of the Running ES Score & Positions of GeneSet Members on the Rank Ordered List

  

| PROBE | DESCRIPTION (from dataset) | GENE SYMBOL | GENE\_TITLE | RANK IN GENE LIST | RANK METRIC SCORE | RUNNING ES | CORE ENRICHMENT || 1 | TNFRSF12A | na |  |  | 1130 | 1.611 | -0.0288 | No |
| 2 | IL3RA | na |  |  | 2111 | 1.235 | -0.0569 | No |
| 3 | CSF2RA | na |  |  | 3308 | 0.942 | -0.1017 | No |
| 4 | TYK2 | na |  |  | 4267 | 0.771 | -0.1373 | No |
| 5 | IL17RA | na |  |  | 5175 | 0.625 | -0.1730 | No |
| 6 | CD44 | na |  |  | 5293 | 0.607 | -0.1677 | No |
| 7 | JUN | na |  |  | 5642 | 0.555 | -0.1755 | No |
| 8 | MAP3K8 | na |  |  | 6069 | 0.493 | -0.1885 | No |
| 9 | STAT2 | na |  |  | 6213 | 0.474 | -0.1871 | No |
| 10 | IL7 | na |  |  | 7239 | 0.338 | -0.2343 | No |
| 11 | PTPN2 | na |  |  | 8085 | 0.230 | -0.2741 | No |
| 12 | PTPN1 | na |  |  | 9696 | 0.050 | -0.3573 | No |
| 13 | TNFRSF1B | na |  |  | 9814 | 0.033 | -0.3628 | No |
| 14 | HMOX1 | na |  |  | 10412 | -0.037 | -0.3933 | No |
| 15 | IL15RA | na |  |  | 10423 | -0.038 | -0.3931 | No |
| 16 | PDGFC | na |  |  | 10630 | -0.057 | -0.4028 | No |
| 17 | TLR2 | na |  |  | 10870 | -0.089 | -0.4136 | No |
| 18 | IRF1 | na |  |  | 11060 | -0.115 | -0.4213 | No |
| 19 | PIM1 | na |  |  | 11181 | -0.119 | -0.4254 | No |
| 20 | LTBR | na |  |  | 11263 | -0.132 | -0.4271 | No |
| 21 | STAT1 | na |  |  | 11412 | -0.148 | -0.4321 | No |
| 22 | HAX1 | na |  |  | 11542 | -0.167 | -0.4357 | No |
| 23 | CBL | na |  |  | 11708 | -0.187 | -0.4408 | No |
| 24 | GRB2 | na |  |  | 12300 | -0.261 | -0.4668 | No |
| 25 | IL4R | na |  |  | 12312 | -0.263 | -0.4624 | No |
| 26 | SOCS3 | na |  |  | 12654 | -0.306 | -0.4745 | No |
| 27 | MYD88 | na |  |  | 12655 | -0.306 | -0.4688 | No |
| 28 | BAK1 | na |  |  | 12757 | -0.324 | -0.4680 | No |
| 29 | IL17RB | na |  |  | 13275 | -0.382 | -0.4878 | No |
| 30 | PTPN11 | na |  |  | 13310 | -0.388 | -0.4823 | No |
| 31 | FAS | na |  |  | 13787 | -0.456 | -0.4986 | No |
| 32 | IRF9 | na |  |  | 13794 | -0.458 | -0.4904 | No |
| 33 | TGFB1 | na |  |  | 14260 | -0.515 | -0.5050 | No |
| 34 | TNFRSF21 | na |  |  | 14534 | -0.561 | -0.5088 | No |
| 35 | TNF | na |  |  | 14846 | -0.611 | -0.5135 | No |
| 36 | CXCL10 | na |  |  | 14952 | -0.628 | -0.5073 | No |
| 37 | EBI3 | na |  |  | 15273 | -0.684 | -0.5111 | No |
| 38 | A2M | na |  |  | 15638 | -0.749 | -0.5161 | No |
| 39 | TNFRSF1A | na |  |  | 16016 | -0.823 | -0.5204 | Yes |
| 40 | ACVR1B | na |  |  | 16066 | -0.837 | -0.5072 | Yes |
| 41 | CSF2 | na |  |  | 16092 | -0.842 | -0.4927 | Yes |
| 42 | STAT3 | na |  |  | 16112 | -0.847 | -0.4778 | Yes |
| 43 | CD14 | na |  |  | 16500 | -0.948 | -0.4803 | Yes |
| 44 | CXCL1 | na |  |  | 16603 | -0.969 | -0.4674 | Yes |
| 45 | IFNAR1 | na |  |  | 16604 | -0.970 | -0.4492 | Yes |
| 46 | IL10RB | na |  |  | 16720 | -1.004 | -0.4364 | Yes |
| 47 | CRLF2 | na |  |  | 16928 | -1.063 | -0.4273 | Yes |
| 48 | CXCL3 | na |  |  | 17374 | -1.206 | -0.4279 | Yes |
| 49 | LEPR | na |  |  | 17393 | -1.212 | -0.4061 | Yes |
| 50 | STAM2 | na |  |  | 17437 | -1.229 | -0.3853 | Yes |
| 51 | SOCS1 | na |  |  | 17552 | -1.279 | -0.3672 | Yes |
| 52 | IL13RA1 | na |  |  | 17613 | -1.300 | -0.3460 | Yes |
| 53 | IL1R1 | na |  |  | 18099 | -1.548 | -0.3423 | Yes |
| 54 | IL1B | na |  |  | 18115 | -1.556 | -0.3139 | Yes |
| 55 | IL6 | na |  |  | 18227 | -1.619 | -0.2893 | Yes |
| 56 | IFNGR2 | na |  |  | 18361 | -1.722 | -0.2639 | Yes |
| 57 | IL6ST | na |  |  | 18442 | -1.792 | -0.2345 | Yes |
| 58 | OSMR | na |  |  | 18741 | -2.102 | -0.2106 | Yes |
| 59 | LTB | na |  |  | 18802 | -2.196 | -0.1725 | Yes |
| 60 | IFNGR1 | na |  |  | 18912 | -2.376 | -0.1337 | Yes |
| 61 | ITGB3 | na |  |  | 18981 | -2.535 | -0.0896 | Yes |
| 62 | CD9 | na |  |  | 19017 | -2.631 | -0.0421 | Yes |
| 63 | CXCL11 | na |  |  | 19056 | -2.745 | 0.0074 | Yes |
Table: GSEA details [plain text format]

  

Fig 2: HALLMARK\_IL6\_JAK\_STAT3\_SIGNALING      
 Blue-Pink O' Gram in the Space of the Analyzed GeneSet

  

Fig 3: HALLMARK\_IL6\_JAK\_STAT3\_SIGNALING: Random ES distribution      
 Gene set null distribution of ES for **HALLMARK\_IL6\_JAK\_STAT3\_SIGNALING**
